# The efficacy of lymphaticovenular anastomosis in breast cancer-related lymphedema

**DOI:** 10.1007/s10549-017-4335-0

**Published:** 2017-06-12

**Authors:** H. Winters, H. J. P. Tielemans, M. Hameeteman, V. A. A. Paulus, C. H. Beurskens, N. J. Slater, D. J. O. Ulrich

**Affiliations:** 1 Department of Plastic and Reconstructive Surgery, Radboud University Medical Center, 9101, 6500 HB Nijmegen, The Netherlands; 2 Department of Orthopaedics, Section of Physical Therapy, Radboud University Medical Center, 9101, 6500 HB Nijmegen, The Netherlands

**Keywords:** Lymphaticovenular anastomosis, Lymphedema surgery, Lymphedema treatment

## Abstract

**Introduction:**

Lymphedema can be a debilitating condition, causing a great decrease in a person’s quality of life (QoL). Treatment with lymphaticovenular anastomosis (LVA), in which an anastomosis is created between the lymphatic and venous system, may attenuate lymphedema symptoms and reduce swelling. In this study, we share the results using LVA to treat breast cancer-related lymphedema (BCRL) at our institution.

**Materials and methods:**

Patients were eligible for inclusion if they suffered from unilateral BCRL, if functional lymphatics were available, if compression therapy was used for at least 6 months, and if the follow-up was 12 months at minimum. Lymph vessel functionality was assessed preoperatively using indocyanine green (ICG). During surgery, 1–3 anastomoses were created and shunt patency was confirmed using ICG. Arm volumes were measured before surgery and at 6- and 12-month follow-up. QoL was measured before surgery and at 6-month follow-up. Arm volume differences between the healthy arm and affected arm were compared between the time points.

**Results:**

Twenty-nine consecutive female patients with unilateral BCRL were included. The preoperative mean difference in arm volumes was 701 ± 435 ml (36.9%). This was reduced to 496 ± 302 ml (24.7%) at 6-month follow-up (*p* *=* 0.00). At 12-month follow-up, the mean difference in arm volume was 467 ± 303 ml (23.5%) (*p* *=* 0.02). The overall perceived QoL was increased from 5.8 ± 1.1 to 7.4 ± 0.7 (*p* *=* 0.00). The functionality score decreased from 2.2 to 1.8 (*p* *=* 0.00), the appearance score decreased from 2.6 to 1.9 (*p* *=* 0.00), the symptoms score decreased from 2.8 to 1.8 (*p* *=* 0.00), and the mood score decreased from 2.7 to 1.5 (*p* *=* 0.00). Fifteen patients (53.6%) were able to discontinue the use of compression garment.

**Conclusion:**

Treatment with LVAs is effective in reducing arm volume difference in patients suffering from BCRL. Although no complete reduction of the edema was achieved at 12-month follow-up, the procedure significantly increased the patients’ QoL.

## Introduction

Lymphedema can be a debilitating condition, causing pain, body image disturbances, frequent infections, restrictions in range of motion, and a great decrease in a person’s quality of life (QoL) [1]. Axillary lymph node dissection, radiation therapy to the axillary region, postoperative seroma in the axillary region, and obesity are major risk factors for the development of lymph edema [2]. Reports on the incidence of lymphedema following breast cancer treatment vary widely with 24–49% following mastectomies and 4–28% following breast conserving therapy [3]. When the swelling of a lymphedematous extremity is due to excess fluid, like in the earlier stages of lymphedema progression, pitting edema can be observed. When the excess volume is due to adipose or fibrous tissue, pitting edema will be minimal or absent [4].

Treatment of lymphedema consists of both non-operative and operative methods [5]. Conservative treatment is currently considered to be the standard of care. This includes lymphatic-specific massage techniques, exercise, and external compression. The goal of the treatment is to manually compress tissue and to remove the retained interstitial fluid [6]. After this, the fitted garments are required to prevent the re-accumulation of fluid. However, this therapy is primarily aimed at delaying progression and is not curative [7].

Surgical treatments for lymphedema mainly focus on excisional and reconstructive techniques. Excisional surgery includes debulking and liposuction [8]. Reconstructive options include lymphaticovenular anastomosis (LVA), lymphovenous-lymphatic (LVL) transplant, lymphatic vessel transplantation, and vascularized lymph node transfer (VLNT). [3, 9–11]. Currently, reconstructive options are considered to be more effective in early-stage ‘pitting’ lymphedema due to the progressive nature of this condition. In later stages, when there is non-pitting lymphedema, reconstructive options may not be viable due to the absence of functional lymphatics [12].

Using LVA, the lymph fluid in the extremity affected by lymphedema can bypass the natural route of traveling through lymph vessels to the subclavian veins and entering the bloodstream. This technique was first described in 1963 by Laine and Howard in a rat model [13]. In 1969, Yamada performed studies on LVA in dogs. Several other authors have, since then, improved this procedure so it could be utilized in the treatment of lymphedema in humans [9]. To allow for lymphatic fluid to enter the venous blood stream through a LVA, it is important that the pressure in the lymphatic system is higher than the pressure in the recipient vein. Since there may be a lower pressure in smaller venules compared to larger veins, utilizing small venules as recipient vessels might lower the risk of occlusion of the LVA due to venous backflow [14–16].

With the availability of superfine monofilament sutures and, more recently, indocyanine green (ICG) lymphography, performing LVAs on small subdermal venules and functional lymphatic collectors as small as 0.3 mm has become a practical reality. Recently, promising results regarding lymphedema volume reduction are emerging [17–19]. In addition, previous research demonstrates a significant increase in QoL in patients suffering from BCRL when treated with LVA [20]. In this study, we share the preliminary results using LVA to treat breast cancer-related lymphedema (BCRL) at our institution.

## Patients and methods

### Patients

**Fig. 1:**
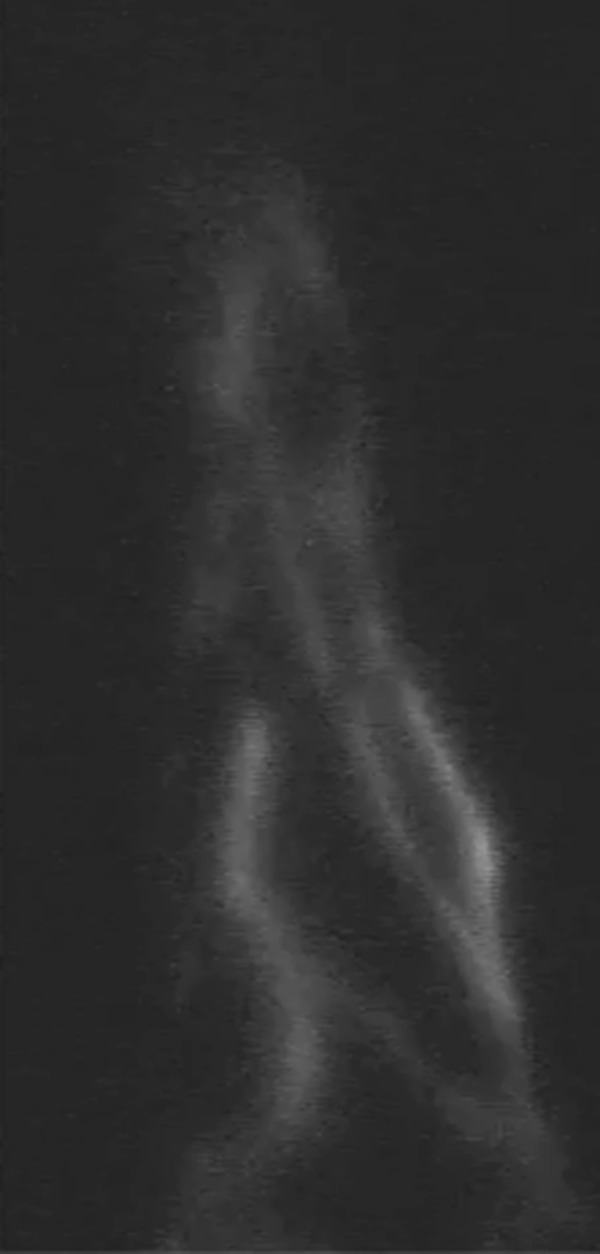
Linear pattern demonstrated by ICG lymphography in a patient with BCRL. This pattern indicates that the lymphatics possess contractility

Patients were eligible for inclusion if they suffered from unilateral BCRL, if functional lymphatics were available, if compression therapy was used for at least 6 months, and if the follow-up was 12 months at minimum. Lymphedema was defined as a volume increase of ≥10% compared to the non-affected arm or self-reported heaviness or swelling, which is the commonly used definition of lymphedema [21]. No limits were set on the duration of the lymphedema. The lymphedema was staged with Campisi’s lymphedema classification [22]. Patient characteristics and baseline volume measurements were noted in a pre-defined form. Lymphatic functionality was evaluated preoperatively using ICG lymphography. For this technique, ICG (0.5%, 0.5 ml, Diagnogreen, Daiichi Pharmaceutical, Tokyo, Japan) was injected subcutaneously in the 2nd webspace of the hand. A photodynamic eye (PDE) was used to identify lymph vessels. Lymphatics were considered functional if ICG lymphography demonstrated a linear pattern according to the Yamamoto ICG staging system (Fig. 1) [23].

### Surgical Technique

Surgery was performed by two experienced plastic surgeons under general anesthesia. Lymphaticovenular anastomoses were performed through 3–4 cm incisions at the distal wrist or forearm in the lymphedematous extremity using a surgical microscope (ZEISS OPMI 800; ×25 to ×50 magnification). The subdermal region was dissected to identify lymphatic vessels of 0.3–0.8 mm in diameter. This was achieved by using ICG lymphography intraoperatively. Similarly sized adjacent venules were explored to anastomose the vessels and create the LVA. LVAs were generally performed end-to-end using 11-0 nylon sutures. If the recipients’ veins were substantially larger, end-to-side anastomoses were performed. Patency of the newly formed anastomosis was confirmed intraoperatively by ICG lymphography. Patients were given a prophylactic antibiotic intraoperatively and for 5 days postoperatively. All patients were discharged within 24 h. After surgery, the affected arm was wrapped with a special compression bandage (Rosidal TCS, Lohmann & Rauscher, Germany) for 1 week and elevated on a pillow. One week after surgery, patients started to continue previous compression therapy which included the usage of compression arm sleeves. After 6 months, the possibility to discontinue compression stockings was evaluated on patient request.

### Outcomes

All data were collected according to a standardized protocol at our institution. Therefore, volume measurements and QoL scores were available for each patient at pre-determined time points. The outcomes were collected during chart review in a retrospective fashion.

The primary outcome was the percentage reduction in volume difference between the affected and the healthy arm. Volume measurements of both the lymphedema and healthy extremity were performed using the water displacement technique preoperatively and at 6-, and 12-month follow-up. All measurements were performed by an experienced physiotherapist (CB) using a standardized volumeter and lukewarm water. Previous research indicates that water temperatures varying between 20° and 32° do not affect arm volume [24].

Secondary outcomes were as follows: the change in QoL after 6 months of follow-up, the possibility to discontinue compression garment usage after 6 months of follow-up, and the relation between decrease in volume difference between extremities and the volume decrease of the affected extremity. QoL was measured preoperatively and 6 months after LVA surgery using the LymphQoL arm questionnaire, a validated questionnaire for patients with lymphedema of the arm to determine QoL [25]. In this questionnaire, patients rated their overall QoL (range 1–10) in addition to subdomains regarding functionality, appearance, symptoms, and patients mood (range 1–4). The effect of the affected extremity on these subdomains was scored from one to four. One indicating the swollen extremity affected the QoL in this domain not at all, two a little, three quite a lot and four a lot. An increase in the overall QoL reflects a positive change in the QoL, while a decrease in the subdomains indicates that the subdomain is less affected by the lymphedema. Furthermore, the relations between the preoperative variables, i.e., difference in arm volumes, BMI, and volume difference reduction and increase of QoL, were explored.

### Statistical analysis

Paired student t-tests were used to compare the volume changes between the affected and the healthy extremity before surgery, 6 months of follow-up, and 12 months of follow-up. Then the percentage decrease in arm volume difference was calculated. In addition, paired student t-tests were used to compare the LymohQoL arm questionnaire results before surgery and after 6 months of follow-up. A Pearson’s correlation analysis was performed to determine the correlation between the decrease in volume difference between arms and the decrease in volume of the affected arm only. Correlations between the percentage arm volume difference decrease after 12 months and arm volume difference at baseline, BMI, duration of edema, and number of anastomoses created were calculated.

The increase in QoL was defined as minor or major improvement. Minor improvement was defined as one point increase in the QoL. Major improvement was defined as >2 point increase in the QoL. Then student t-tests were performed to detect differences between minor and major QoL for the variables: arm volume difference at baseline, BMI, duration of edema, and number of anastomoses created. When a significant difference of means was detected, a binary logistic regression was performed to determine the odds for major QoL improvement. All analyses were performed with IBM SPSS version 22 (IBM Corp., Armonk, N.Y.).

## Results

Twenty-nine consecutive female patients with unilateral BCRL were eligible for inclusion. Patient characteristics are listed in Table 1. The mean age of these patients was 59 ± 9 years (range 41–84 years). Their BMI was 27 ± 4 kg/m^2^ (range 21–34). Twelve patients had lymphedema in their left arm and 17 in their right arm. Three of the treated patients had less than 10% volume surplus but experienced complaints because of the edema nonetheless. All patients demonstrated pitting lymphedema (stage 1b–2a according to Campisi). Lymphedema was present for a mean period of 9 ± 7.3 years (range 2–39 years). All patients gave informed consent regarding the surgical procedure and ICG injection.

**Table 1:** Patient characteristics

Patient characteristic	*N* (%)
Age, years, mean (range)	57 (25–84)
BMI, kg/m^2^, median (range)	26 (23–34)
Lymph edema duration, mean (range)	9 (2–39)
Right extremity	16 (59)
Anastomoses	53 (100)
End-to-end	45 (84)
End-to-side	6 (12)
Invagination	2 (4)
Number of anastomosis, mean (range)	1.8 (1–3)
Operative time, min, mean	168

The mean number of anastomoses was 1.8 ± 0.8 (range 1–3). Ten patients received 1 LVA, 14 patients two LVAs, and five patients three LVAs. The anastomoses were most commonly performed end-to-end (*n* = 45), followed by end-to-side (*n* = 6), and the invagination technique (*n* = 2). The diameter of the lymphatic vessels used for bypass ranged from 0.3 to 0.7 mm. The mean operative time was 2.8 ± 0.4 h. No postoperative complications, defined as complication occurring within 30 days after surgery, occurred. During follow-up, two patients endured two episodes of cellulitis.

### Volume measurements

**Fig. 2:**
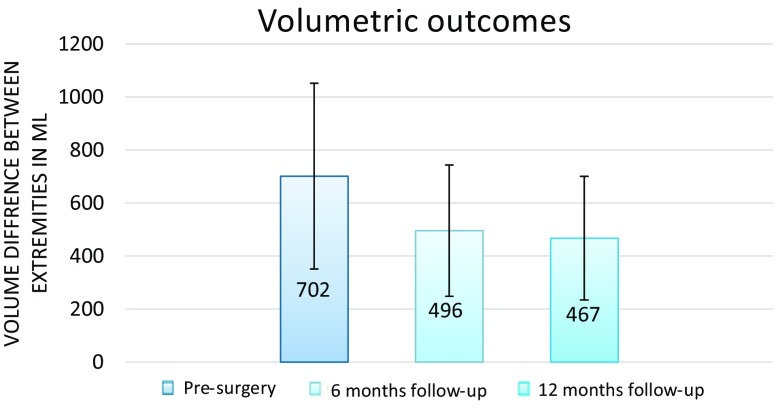
Average volume differences between the healthy and affected extremity at different time points during follow-up. Error bars indicate standard deviation (SD). Significance was reached between baseline and 6 months of follow-up (*p* < 0.001), between baseline and 12 months of follow-up (*p* < 0.001), and between 6 months of follow-up and 12 months of follow-up (*p* < 0.02)

The preoperative mean difference in arm volume was 701 ± 435 ml. This was reduced to 496 ± 302 ml at 6-month follow-up (*p* < 0.001). At 12-month follow-up, the mean difference in arm volume was 467 ± 303 ml (23.5%) (*p* < 0.001) (Fig. 2). Therefore, the percentage volume reductions at 6- and 12-month follow-up were 29 and 33%, respectively. Of the 29 patients, 28 patients treated with LVA showed an improvement of the volume difference between arms. The volumetric arm difference increased in one patient. When the decrease of arm volume difference between the affected and the healthy extremity was compared to the decrease of volume of the affected extremity only, the correlation was *r* = 0.60 (*p* *=* 0.00)

There was no significant correlation between the volume difference reduction between arms and the variables: arm volume difference at baseline (*r* = −0.15, *p* *=* 0.44), BMI (*r* = −0.06, *p* *=* 0.52), duration of edema (*r* = −0.15, *p* *=* 0.45), number of anastomoses created (*r* = −0.03, *p* *=* 0.89).

### Quality of life

**Fig. 3:**
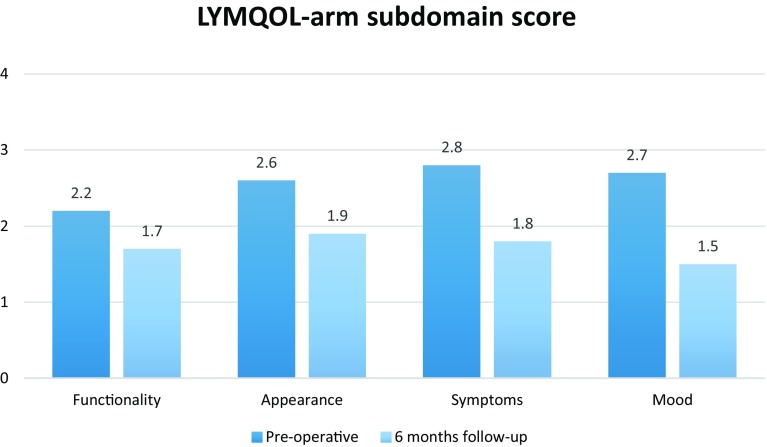
Mean reduction and standard error (SE) of the LYMQOL-arm index score regarding the subdomains. A lower score in the subdomains indicates that patients were less affected by the lymphedema in that subdomain

The overall perceived QoL was increased from 5.8 ± 1.1 to 7.4 ± 0.7 (*p* *=* 0.00). The functionality score decreased from 2.2 to 1.8 (*p* *=* 0.00), the appearance score decreased from 2.6 to 1.9 (*p* *=* 0.00), the symptoms score decreased from 2.8 to 1.8 (*p* *=* 0.00), and the mood score decreased from 2.7 to 1.5 (*p* *=* 0.00) (Fig. 3). Fifteen patients (53.6%) were able to discontinue the use of compression garment.

Considering minor versus major improvement in QoL, only the arm volume difference at baseline was significantly higher in the major QoL improvement group. Arm volume difference at baseline was 473 ml in the minor improvement group and 907 ml in the major improvement group (*p* *=* 0.007). The mean BMI was 25.8 in the minor improvement group and 28.6 in the major improvement group (*p* *=* 0.06). Mean duration of edema was 10.3 years in the minor improvement group and 8.07 years in the major improvement group (*p* *=* 0.45). The mean amount of anastomoses created was 2.08 in the minor improvement group and 1.60 in the major improvement group (*p* *=* 0.08).

The variable arm volume difference at baseline was categorized in groups in which the arm volume difference was increased by 250 ml each step. Then a binary logistic regression analysis was performed. The odds ratio (OR) to a major increase in QoL was 2.06 (*p* *=* 0.02, CI = 1.10–3.86) per 250 ml.

## Discussion

Although LVA was considered a controversial technique in the treatment of lymphedema, it is gaining popularity with the advancement of microsurgical techniques. Previous studies demonstrate mixed results following the LVA procedures, but the quality of these studies vary. In addition, most studies evaluating LVA present the results in primary and secondary lymphedema of both the upper and lower extremities as one, while the effect of LVA most likely differs per lymphedema modality [26, 27].

This study demonstrates the results of LVA surgery in secondary lymphedema resulting from breast cancer treatment. To our knowledge, this study is the first to assess the effects of LVA on the QoL of BCRL patients who experienced significant volume reduction between arms. In addition, although in a relatively small population, it is the first time the relation between preoperative variables and outcomes is explored.

For this study, only patients with unilateral BRCL were included. This allowed for the unaffected arm to be used as inpatient control. Patients included in this study experienced a reduction of 33% of the arm volume difference at 12-month follow-up. In one patient, the volume difference between arms increased, but the QoL increased too. The QoL of patients increased in all but one patient. In this patient, there was a volume decrease between arms. The preoperative arm volume difference proved to be a significant predictor to a greater increase of QoL. Interestingly, the decrease in arm volume difference between arms was not larger in patients who experienced a greater increase in QoL. In this study, the possible volume effect of arm dominancy was not taken into account.

In our study population, the patients' BMI, the amount of shunts created, and the duration of the edema did not affect the effect of the procedure on volume reduction between arms or QoL. While the QoL was increased in the majority of patients after 6 months, it is likely that the QoL was further increased after 6 months due to the great amount of patients that were able to discontinue the use of compression garment.

Since lymphedema surgery is considered controversial in the Netherlands and its place in the treatment of lymphedema is still unconfirmed, we were very cautious concerning the discontinuation of compression garments. In the population studied in this paper, patients came with the request to discontinue compression therapy when they noticed attenuation of their complaints. Only then, we advised to slowly phase the discontinuation of compression garments by increasing the time spent without compression garments. Then, if arm volume remained stable or decreased, and the patient noticed no subjective increase of the lymphedema, the compression therapy could be fully discontinued. For all patients, we strongly encouraged the use of compression therapy until minimally 6 months after surgery.

The results demonstrated in this study are in line with recent trials evaluating LVAs in secondary upper limb lymphedema patients. Chang et al. reported that arm volumes decreased in 74% of patients with upper limb lymphedema, and these patients experienced a volume reduction of 42% of the affected limb [28]. Poumellec et al. found a volume reduction of 22.5, 21.32, and 30.24% in the affected wrist, forearm, and arm, respectively [29]. In our study, the effect of LVAs was determined by comparing the volume differences between the affected and the healthy arm between time points, instead of comparing the difference in volume of the affected arm only. This ensures that the measured results are not affected by variables such as air humidity and temperature and therefore reflects a more precise effect of the procedure. This is underlined by the low correlation between decrease of volume of the affected arm and the decrease of volume difference between arms found in this study (*r* = 0.60).

Volume measurements of both the lymphedematous and healthy extremity were performed using a water displacement technique. This technique is highly accurate in measuring arm volume with an intraclass correlation coefficient (ICC) of 0.99 [30, 31]. More recently, we however started measuring lymphedema volumes with 3D stereo photogrammetry in our institution. This may measure lymphedema volumes more accurate and may become the first choice diagnostic tool for lymphedema volume assessment [32–34]. The effect of limb dominance on arm volume is known to be statistically significant but small and was not taken into account in this study [35].

Concerning the peri-operative care for lymphaticovenular anastomosis, a protocol was followed based on several recommendations in the current literature. It is however imperative to note that these recommendations are solely based on expert opinions and no evidence is available to support these suggestions. Animal studies indicate that the long-term patency of LVAs can be as low as 52%. Therefore, it is of great importance to optimize the peri-operative conditions to improve the shunt patency. As it is currently unknown if peri-operative interventions such as compression therapy directly post-surgery may either harm or benefit the patency, future studies should clearly state the peri-operative care that was used. Then it may become possible to optimize the effect of LVA.

Although the results of this study were analyzed in retrospect, data were collected according to a standardized protocol at our institution. Therefore, data concerning arm volumes were available for each patient at pre-determined times during follow-up. In addition, preoperative variables such as ICG lymphography and duration of the lymphedema were also noted for each patient. To ensure that all anastomoses were patent directly post-surgery, ICG lymphography was used during surgery.

## Conclusion

Treatment of BRCL with LVA seems an effective strategy in reducing the volume difference between arms and increasing the patients QoL. Interestingly, the reduction in volume differences was not correlated to a greater increase in QoL. In addition, the amount of shunts created, patients BMI, and the lymphedema duration did not affect volume reduction or the patients QoL. Future research, most preferably in a randomized controlled fashion, should confirm these findings.
